# Chromatin Organization and Nuclear Architecture as Indicators of Plant Cell Developmental Competence

**DOI:** 10.3390/plants15213419

**Published:** 2026-11-03

**Authors:** Taras Pasternak, Douglas Steinmacher

**Affiliations:** 1 Plantae Consulting ES, 03202 Elche, Spain; 2 Verdant Center for Spatial Biology, 30 N Gould St., Ste N, Sheridan, WY 82801, USA; 3 AlfaPalm Agrociências, Marechal Cândido Rondon 85960-148, PR, Brazil

**Keywords:** 3D nuclear geometry, chromatin compaction, cell-cycle re-entry, protoplast microculture, organogenesis, *Medicago sativa*, *Arabidopsis thaliana*

## Abstract

Plant cell totipotency is a fundamental property of the plant kingdom. Although most plant cells contain essentially identical genetic information, only a subset retains the ability to re-enter the cell cycle, and even fewer keep the ability to regenerate whole plants. One possible explanation is that cells differ in their chromatin organization. Here, we investigated whether 3D nuclear architecture reflected cell-cycle probability and cell-cycle re-entry and totipotent reprogramming. Using confocal profiling across root tissue layers (*n* = 50 nuclei/layer; N ≥ 3 biological replicates) and root protoplast microcultures of *Arabidopsis thaliana* and *Medicago sativa*, we show that nuclear geometry serves as a quantifiable physical proxy(marker/indicator) for cell permissiveness. In *A. thaliana*, non-competent cortical cells face a steep ~78% nuclear volume reduction barrier (>10,000 µm^3^ to ~2500 µm^3^), restricting cell-cycle entry (0%). In *M. sativa*, shallower structural gradients allow cortical cells to divide (>70%), yielding non-regenerative callus. Conversely, pericycle cells maintaining compact nuclear states retain shoot organogenesis (>35%). Uncoupling tissue constraints confirms that cell-cycle re-entry and true totipotency represent two distinct biophysical transitions. Re-establishing whole plants strictly requires transitioning to a low-entropy 3D nuclear state, establishing baseline nuclear geometry as a non-invasive physical indicator of developmental competence. These findings establish baseline nuclear geometry as a non-invasive morphological indicator to distinguish vegetative proliferation from true regenerative competence.

## 1. Introduction

Cellular totipotency is one of the most remarkable properties of plant cells. Haberlandt first proposed this phenomenon (1902) [1], and it was later experimentally demonstrated in the cambium of some plant species through activation of cell division [2,3]. Stewart and colleagues demonstrated the formation of whole new plants in carrot cell cultures [4,5]. Despite sharing essentially the same genome, only a subset of differentiated plant cells can re-enter the cell cycle and regenerate whole plants. The mechanisms underlying this differential developmental competence remain incompletely understood.

Current models have identified numerous hormonal, transcriptional, and epigenetic regulators of plant regeneration [6]. However, why specific somatic cells within an explant retain developmental competence while adjacent cells permanently lose it remains a fundamental question. For instance, localized cell-fate plasticity is well illustrated in the leaf epidermis, where stomatal lineage precursor cells maintain a permissive chromatin architecture and positional identity that enable specialized cell division and fate specification, whereas adjacent mesophyll cells remain terminally constrained [7].

Similarly, in *Arabidopsis* root, only pericycle cells keep the ability to re-enter the cell cycle and support plant regeneration [8].

This tissue-level heterogeneity underscores that developmental competence and ultimately the capacity for totipotent reprogramming are strictly gated by baseline 3D nuclear architecture and cellular context. Chromatin organization is tightly controlled in plant and animal cell development, allowing activation or repression of specific DNA regions [9]. Histone acetylation and DNA methylation control this organization and depend on local hormonal balance and auxin source types; each YUCCA-expressing cell has a unique chromatin organization [8]. Besides histone modification, DNA methylation is another marker of cell biological age; with progressive aging, it significantly increases [10]. DNA methylation occurs in different nucleotide contexts in animal and plant genomes: cytosines in CpG contexts are major targets of DNA methylation in animal genomes, whereas in plants, Cs in all nucleotide contexts are subject to methylation [11]. However, DNA methylation in plants is not a reliable marker, as a competent cell may have DNA methylation similar to that in a stem cell or far from that in differentiated mesophyll cells; in plant cells, DNA methylation is more flexibly controlled, and plants are even able to survive larger reductions in genomic 5mC than mammals, suggesting that DNA methylation is not a control point in plant cell totipotency [9,11,12].

A recent publication described the role of chromatin modification in plant cell dedifferentiation [13]. However, the authors still rely on whole-organ analysis and lack direct in situ evidence, but they pointed out the relevant role of histone (de)acetylation during plant cell totipotency realization. This points to evidence that the WOUND INDUCED DEDIFFERENTIATION 1 (WIND1) transcription factor, which interacts with histone deacetylase, promotes somatic embryogenesis [14]. Importantly, wounding serves as an essential prerequisite for plant in vitro regeneration. In this context, wounding can be interpreted as a physiological trigger that disrupts and redistributes hormonal gradients, particularly auxin polar transport.

Realizing somatic cell totipotency requires activating cell division and switching from the somatic to generative type of cell division. Therefore, the cell cycle needs to be looked at in detail. In planta, the maximum and rapid cell-cycle activity was detected in somatic cells after cell fate establishment [15].

From a physical/chemical point of view, the plant cell cycle is a polymer transition governed not merely by transcription waves but by precise spatial and thermodynamic reorganization of nuclear architecture [16,17]. Rather than serving as a passive template, chromatin organization acts as an upstream topological license that dictates the accessibility and efficiency of the cell-cycle machinery [18,19]. While core cell-cycle regulators (such as CDK/cyclin complexes) provide the biochemical catalytic drive, the spatial chromatin architecture, defined by its 3D folding and local density, establishes the physical permissiveness of the genomic landscape. In highly proliferating, undifferentiated systems, such as suspension cells, chromatin adopts a highly accessible, low-compaction state that offers minimal physical resistance to replication and transcription factors. Conversely, in intact, differentiated somatic cells, cell-type-specific chromatin compaction imposes rigid topological barriers [17,20]. Consequently, the mere abundance of cell-cycle regulators is insufficient to drive replication and mitosis if the local chromatin environment fails to undergo the requisite spatial transitions to license DNA replication and mitosis [21].

This biophysical regulation operates as a thermodynamic state transition, in which chromatin geometry directly influences the cell’s developmental trajectory and transcriptional reprogramming [22]. In this context, somatic cell proliferation (such as callus initiation) typically occurs in cells exhibiting a highly relaxed, high-entropy chromatin organization. While this structural looseness facilitates rapid cell-cycle re-entry, it reduces the constraint on loop-extruding complexes, increasing susceptibility to epigenetic instability, chromatin mixing, and subsequent somaclonal variation. Conversely, the transition to true embryogenic competence requires a highly ordered, low-entropy chromatin configuration. This compact, geometrically structured state ensures high-fidelity transmission of genetic and epigenetic information across cell divisions, allowing coordinated activation of the complex gene regulatory networks required for de novo organogenesis and successful plant regeneration.

In this paper, we propose that chromatin organization is an upstream structural marker of cellular totipotency that can predict cell competence. Cell-cycle re-entry and subsequent embryogenic competence require distinct chromatin states corresponding to two different levels of developmental reprogramming, and somatic cell totipotency consists of two consecutive transitions. The first transition enables differentiated cells to regain mitotic competence and re-enter the vegetative cell cycle. The second transition establishes a stem-cell-like chromatin state that permits de novo organogenesis or somatic embryogenesis. Somatic proliferation generally occurs in cells with more relaxed chromatin organization, which may increase susceptibility to epigenetic instability and contribute to somaclonal variation, particularly following prolonged callus culture. Therefore, the realization of plant cell totipotency for plant regeneration requires two sequential developmental transitions. The first is acquiring mitotic competence, resulting in somatic (callus-type) cell proliferation. The second is the establishment of an embryogenic, stem-cell-like chromatin state that enables organogenesis or somatic embryogenesis. Although both transitions depend on endogenous auxin, they differ fundamentally in chromatin organization and probably also in the underlying auxin biosynthetic pathways. Thus, auxin is essential for cell-cycle activation, but exogenous auxin alone is not sufficient to induce either cell-cycle re-entry or cellular totipotency. In this study, we focus on in situ quantitative cytological and 3D nuclear morphometric profiling. We establish nuclear volume, sphericity, and compaction density as physical proxies that reflect structural genome constraints, providing a biophysical framework that correlates nuclear geometry with cell-cycle licensing and developmental plasticity.

## 2. Results

In the present investigation, we sought to determine whether the spatial geometry and compaction state of chromatin act as active, predictive determinants of cell-cycle re-entry and somatic totipotency in plant tissues. Rather than treating chromatin as a passive template subordinate to transcriptional waves, we hypothesized that the 3D nuclear architecture establishes a physical and topological license that either permits or restricts mitotic reactivation. To test this hypothesis, we performed high-resolution comparative nuclear profiling across the radial tissue layers of *Arabidopsis thaliana* and *Medicago sativa* root explants, which serve as highly characterized, physiologically contrasted model systems of regenerative competence.

To map the spatial distribution of nuclear phenotypes in planta, we utilized 3D confocal laser scanning microscopy (CLSM) coupled with quantitative morphometric analysis of roots stained with propidium iodide (PI) after RNase A digestion. This experimental setup allowed us to selectively visualize genomic DNA geometry and chromatin compaction without interference from nuclear RNA. By profiling tissue segments from the root apical meristem (RAM) to the mature zone, we systematically analyzed nuclear size, shape, and chromatin homogeneity in individual cells within the pericycle, endodermis, and cortical layers.

Our results reveal a striking radial divergence in chromatin organization that directly correlates with cell-fate plasticity and mitotic potential. In both species, pericycle cells maintain a highly ordered, compact, and low-entropy chromatin state, mirroring the nuclear signature of stem-cell-like initials. However, the outer cell layers display divergent patterns: while *A. thaliana* exhibits a steep, rigid radial gradient of chromatin compaction that strictly limits mitotic competence to the pericycle, *M. sativa* displays a significantly shallower gradient, with the inner cortex (Cortex II) retaining a permissive, low-entropy chromatin state. Our protoplast experiments further validate these spatial constraints, showing that uncoupling cells from tissue-level radial inhibition exposes their autonomous, chromatin-determined developmental potential.

### 2.1. Radial Divergence of Chromatin Geometry in Root Apical Meristems (RAM)

To investigate the initial spatial distribution of chromatin states across different cell-type trajectories, we first examined the nuclear architecture within the root tips of both species up to the transition zone (Figure 1). Whole roots were fixed and stained with propidium iodide following RNase A treatment to achieve high-contrast, selective labeling of genomic DNA (Figure 1A,B). High-resolution confocal imaging and subsequent 3D reconstructions revealed a pronounced radial gradient of chromatin compaction and nuclear morphology, demonstrating that distinct tissue layers possess highly specialized nuclear signatures long before final differentiation.

In *Arabidopsis thaliana* (Figure 1A), we observed a steep and highly coordinated radial divergence in chromatin organization. Pericycle nuclei exhibited an extremely stable, uniform, compact geometric conformation, characterized by highly condensed chromocenters and low structural entropy. Moving outward through the endodermis and into the cortical layers, this compact arrangement was progressively lost. Cortical nuclei showed pronounced morphological heterogeneity, a significant increase in nuclear volume, and a highly variable distribution of heterochromatin, indicating a rapid chromatin disorder in these differentiated outer layers (Figure S1).

In contrast, *Medicago sativa* (Figure 1B,B1) exhibited a much shallower radial gradient of chromatin compaction, reflecting a more plastic nuclear state. While pericycle nuclei in *Medicago* maintained a compact, homogeneous geometry similar to *Arabidopsis*, the adjacent cortical layers did not show the same rapid transition to low-order structure. Specifically, nuclei in the inner cortical cells (Cortex II-III) retained a relatively compact, uniform chromatin conformation with well-defined heterochromatin domains (Figure S2). This structural homogeneity in the *Medicago* cortex contrasts sharply with the highly heterogeneous and variable cortical nuclei observed in *Arabidopsis*, suggesting that *Medicago* cortical cells remain epigenetically closer to a competent, low-entropy state permissive for cell-cycle re-entry.

### 2.2. Tissue-Level Gradients Correlate with Strict Division Competence in Planta

To determine how these early chromatin differences affect mature tissue plasticity, we analyzed the spatial and lateral gradients of cell volume and nuclear architecture within the mature root zone of both species (Figure 2). Our three-dimensional (3D) confocal reconstructions revealed a striking divergence in tissue-level gradients between *Arabidopsis thaliana* (Figure 2A) and *Medicago sativa* (Figure 2B). In *Arabidopsis thaliana*, we observed a steep centripetal gradient of both lateral cell volume and chromatin state. While the central vascular and pericycle cells remained small, tightly compact, and structurally ordered, the adjacent cortical and epidermal layers exhibited a rapid, dramatic increase in cell volume and a corresponding loss of chromatin compaction. This steep structural gradient in *Arabidopsis* reflects a rigid physical transition, where the outer cell layers rapidly accumulate nuclear and cellular disorder as they differentiate, establishing a strict spatial boundary that restricts developmental plasticity.

In contrast, *M. sativa* exhibited a significantly shallower radial gradient of cell volume and nuclear compaction across the mature root tissues (Figure 2B). Quantifying the cell and nuclear dimensions from the central vasculature to the outer epidermis revealed that the inner cortical cells (specifically Cortex II_III) of *Medicago* maintain a highly conserved, compact morphology, with significantly smaller cell volumes compared to the highly expanded cortical layers of *Arabidopsis*. This structural preservation correlates with a lower baseline of chromatin entropy, indicating that *Medicago* cortical cells do not undergo the same terminal epigenetic lock observed in *Arabidopsis*. Consequently, when exposed to exogenous mitotic cues, these low-entropy inner cortical cells in *Medicago* remain highly permissive to cell-cycle reactivation, whereas in *Arabidopsis*, the steep physical and epigenetic barrier restricts mitotic re-entry strictly and exclusively to the pericycle layer.

These divergent tissue gradients directly dictate the downstream regenerative trajectories and morphogenetic outcomes of both species under hormonal reprogramming. In *Arabidopsis*, where division competence is confined to the pericycle, treatment with a two-step regeneration protocol, consisting of initial auxin priming (2,4-D) followed by cytokinin-induced shoot promotion (BAP), leads to highly coordinated, direct, or semi-direct shoot organogenesis from the pericycle-derived founder cells. Because these cells initiate division from a highly ordered, low-entropy state, they successfully maintain the epigenetic memory required for de novo organ patterning. Conversely, in *Medicago*, the high mitotic permissiveness of the Cortex II-III cells results in extremely rapid, unorganized periclinal divisions upon 2,4-D exposure. This explosive proliferation quickly forms a soft, rapidly expanding callus from the outer root layers. However, because these cortical divisions bypass the highly structured, stem-cell-like chromatin configuration of the pericycle, the resulting callus tissue lacks shoot regeneration competence in intact explants, illustrating a fundamental developmental uncoupling between rapid vegetative cell division and true somatic totipotency.

### 2.3. Quantitative Analysis

To evaluate potential variations in structural barriers during chromatin state transitions, we compared the remodeling barrier required to transition from a cortical to a pericycle nuclear type in *Arabidopsis thaliana* and *Medicago sativa* (Figure 3).

Compared to *A. thaliana*, cortex and pericycle nuclei in *M. sativa* exhibit substantially less structural divergence, particularly regarding nuclear volume and surface area. This reduced separation suggests that acquiring a cell division-competent nuclear state in *M. sativa* requires less structure-associated remodeling, consistent with a lower structural barrier to competence. Nevertheless, the pronounced shifts in surface-to-volume ratio and sphericity demonstrate that this transition still entails a meaningful reorganization of nuclear architecture.

We next examined cell-cycle competence in both species by monitoring EdU incorporation during a 6-h labeling period. In *Medicago*, cortical cells adjacent to the endodermis (CII and CIII) readily incorporated EdU, indicating that these cells retain the capacity to enter or progress through S phase (Figure S3). In contrast, EdU incorporation in *Arabidopsis* was largely restricted to the vascular/pericycle region, with differentiated cortical nuclei showing little or no detectable replication activity (Figure S4). Together with the differences in nuclear architecture, these observations reveal an association between nuclear structural organization and replication competence, suggesting that acquisition or maintenance of an appropriate nuclear/chromatin state may be important for cell-cycle progression.

### 2.4. Exogenous Auxin Selectively Triggers Rapid Cortical Proliferation and Callus Initiation in Medicago, but Not in Arabidopsis

To evaluate the physiological consequences of these divergent tissue-level nucleus geometry gradients, we analyzed the morphogenetic response of *Arabidopsis thaliana* and *Medicago sativa* root explants to different exogenous auxin treatments. At a physiological concentration of a natural auxin analog (100 nM NAA), both species showed organized cell divisions restricted to the pericycle, leading to predictable development of lateral root primordia (LRP). However, when explants were exposed to the strong synthetic auxin 2,4-dichlorophenoxyacetic acid (1 mg/L 2,4-D), the two species exhibited highly contrasting developmental trajectories. This divergence highlights how the baseline physical constraints of the tissue dictate how cells perceive and translate external hormonal cues into either organized organogenesis or unorganized proliferation.

In *M. sativa*, the shallower radial gradient of chromatin compaction and cell volume allows outer tissue layers to remain highly responsive to mitotic stimulation. With 1 mg/L (approx. 4.5 µM) 2,4-D, the outer cortical layers of *Medicago* roots underwent rapid periclinal cell divisions. Within only 3 to 4 days after initiation (Figure 4), this explosive cortical proliferation erupted through the epidermis, forming a highly hydrated, soft callus tissue. This rapid response demonstrates that the cortical cells of *Medicago*, which maintain a relatively low-entropy chromatin state in planta, are morphologically and epigenetically primed for rapid cell-cycle re-entry, bypassing the strict spatial restriction observed in *Arabidopsis*.

Conversely, in *A. thaliana*, the steep radial chromatin barrier successfully insulated the cortical layers from 2,4-D-induced mitogenesis, restricting proliferative reactivation exclusively to the pericycle. This localized activation in *Arabidopsis* is clearly illustrated within 36 h of 2,4-D treatment (Figure 5), where mitotic reactivation is strictly confined to the pericycle layer (Figure 5A), while the outer cortical cells remain completely quiescent. High-magnification details (Figure 5B) confirm that the dense, localized divisions within the pericycle form the structural foundation of a compact, organogenic callus.

These comparative results provide strong empirical support for our core hypothesis: cell division competence and developmental totipotency are fundamentally uncoupled processes. This allows us to distinguish between division without developmental reprogramming (as observed in the rapid, unorganized proliferation of the *Medicago* cortex to callus) versus division coupled to a major change in developmental potential (as seen in the pericycle-driven organogenic path of *Arabidopsis*). While a relaxed, permissive chromatin state in the *Medicago* cortex allows rapid cell division and soft callus formation, these cells lack the highly ordered, low-entropy chromatin architecture required for de novo organogenesis. Therefore, somatic totipotency is not merely a consequence of active cell division but depends strictly on the donor cell’s initial, highly structured epigenetic state.

### 2.5. Realization of Totipotency Potential

Next, we checked the realization of the second step of totipotency—de novo shoot induction. In the case of *Arabidopsis*, massive de novo shoot formation was detected after 7 days on the medium with 1 mg/L BAP (Figure S5). However, in *Medicago* roots, mainly callus formed even in the light and in the same medium containing 1 mg/L BAP.

### 2.6. Uncoupling Radial Tissue Constraints via Protoplast Isolation Reveals Cell-Intrinsic Epigenetic Potential

While analysis of intact root explants highlighted the role of radial tissue gradients in restricting cell division, these systems cannot easily distinguish between cell-autonomous programming and tissue-level lateral inhibition. To decouple individual cell potential from cell-to-cell communication and physical tissue constraints, we isolated cell-type-specific protoplasts from *Arabidopsis thaliana* and *Medicago sativa* roots (Figure 6 and Figure 7). Protoplasts serve as an ideal single-cell model system, effectively ‘resetting’ the physical tissue environment by digesting the cell wall and disrupting symplastic connections (Figure 6A–D). This approach allowed us to determine whether cell division competence and developmental totipotency are driven by cell-intrinsic chromatin signatures or are continuously imposed by the surrounding tissue niche.

Our results in *M. sativa* show that once uncoupled from tissue-level constraints, both pericycle and cortical (Cortex II-III) cells can enter the cell cycle (Figure 7). In intact roots, division is predominantly restricted to Cortex II cells, which rapidly form a soft callus (Figure 7A). Following protoplast isolation, both cortex- and pericycle-derived single cells successfully initiated mitotic division in microculture (Figure 7B,C). This critical finding indicates that mitotic inactivity in certain cell layers in planta is not due to an absolute physiological block but is instead regulated by radial tissue-level inhibitions. Once these external constraints are enzymatically removed, the intrinsic machinery for cell-cycle re-entry is unlocked in both differentiated somatic and generative cell types.

However, despite their shared capacity for cell division, the downstream developmental trajectories of the isolated cells remained strictly divergent and cell-autonomous, determined by their initial chromatin states. Protoplasts derived from the inner cortex (Cortex II-III), which exhibit a larger initial cell volume (Figure 7D) and a relaxed, high-entropy chromatin state, underwent rapid but unorganized cell divisions, yielding only soft, non-regenerative callus tissue. Conversely, pericycle- and apex-derived protoplasts, characterized by their small cell volume (Figure 7D) and a highly compact, low-entropy chromatin configuration, preserved their developmental memory. These pericycle-derived cultures established organized division patterns (Figure 7B), leading to embryogenic calli and subsequent de novo shoot organogenesis. These single-cell assays confirm that while cell-cycle re-entry can be generalized upon tissue uncoupling, true somatic totipotency is a cell-intrinsic property predicted by the initial, highly ordered spatial geometry of the chromatin.

## 3. Discussion

### 3.1. Chromatin Geometry as an Upstream Topological License for the Cell Cycle (And Plant Regeneration)

We propose that the eukaryotic cell cycle is driven not merely by transcriptional waves but by a precise spatial, geometric, and thermodynamic transition of the nuclear architecture [23,24]. Rather than serving as a passive template, chromatin organization acts as an upstream topological license that dictates the accessibility and efficiency of cell-cycle machinery [19,21]. While core cell-cycle regulators (such as CDK/cyclin complexes) provide the biochemical catalytic drive, the spatial chromatin architecture—defined by its 3D folding and local compaction—establishes the physical permissiveness of the genomic landscape [23,25]. Specifically, the spatial “geometry” of the genome is dynamically sculpted throughout the cell cycle by structural maintenance of chromosomes (SMC) protein complexes [19]. Cohesin and condensins function as loop-extrusion motors that establish chromosome territories in interphase and mold highly compacted loop arrays during mitotic chromosome folding, thereby dictating nuclear geometry across cell-cycle phases [19]. This three-dimensional conformation acts as a direct regulator of cell-cycle progression and hormone signaling [21,22]. For instance, high-resolution Hi-C mapping in *Brassica napus* empirically demonstrated that dynamic switching between active ‘A’ compartments (euchromatin) and repressed ‘B’ compartments (heterochromatin) directly regulates the expression of key hormone biosynthesis and cell-cycle genes, such as CYCB1 and E2FA/B, which in turn accelerates cell division and drives somatic expansion [26]. This biophysical regulation explains our observations in root apical meristems and mature zones. The steep radial gradient of chromatin compaction in *Arabidopsis thaliana* imposes rigid physical barriers in the outer cortical layers, rendering them insulated from mitotic cues despite the presence of systemic hormones [21,25].

Conversely, the shallower tissue gradient in *Medicago sativa* allows the inner cortex (Cortex II-III) to maintain a highly permissive, low-entropy chromatin configuration, facilitating rapid mitotic re-entry under exogenous auxin stimulation [27,28].

### 3.2. Uncoupling Tissue-Level Radial Constraints to Expose Cell-Autonomous Epigenetic Potential

A major challenge in plant developmental biology is distinguishing between tissue-enforced lateral inhibition and cell-autonomous epigenetic memory [29]. In intact root explants, the complex cellular microenvironment and polar auxin transport mask the true regenerative potential of individual cells [28]. By utilizing isolated protoplast systems, we effectively bypassed these tissue-level radial constraints, allowing us to evaluate the developmental potential of single somatic cells in a “cell-zero” environment [27]. Upon enzymatic cell-wall removal and the disruption of symplastic connections, both cortical and pericycle cells of *Medicago sativa* successfully re-entered the cell cycle, confirming that mitotic competence is a generalized response when cells are liberated from tissue-level inhibition [27]. However, their subsequent developmental trajectories remained strictly divergent and cell autonomous. Cortical protoplasts, which inherit a relaxed, high-entropy chromatin state in planta, underwent rapid but unorganized cell divisions, yielding only non-regenerative, soft callus tissue. In contrast, pericycle- and apex-derived protoplasts, which maintain a compact, low-entropy chromatin configuration, preserved their developmental memory and successfully regenerated shoots. This demonstrates that while cell-cycle re-entry can be triggered by relieving tissue-level constraints, true embryogenic competence is an intrinsic, chromatin-determined property that cannot be artificially imposed by exogenous hormones alone [28].

### 3.3. A Biophysical Two-Stage Model of Somatic Reprogramming: Callus vs. Shoot Competence

Based on these cytological observations, we propose a conceptual two-stage biophysical hypothesis for somatic reprogramming. Within this framework, cell-cycle re-entry (somatic proliferation) and shoot regeneration competence (true totipotency) represent distinct levels of structural organization and epigenetic licensing [28]. Rather than measuring absolute thermodynamic quantities, we utilize nuclear volume, surface-to-volume ratio, sphericity, and compaction density as quantifiable physical proxies reflecting the structural state of the genome. The first transition (vegetative cell-cycle re-entry) is associated with an intermediate-to-relaxed chromatin state that physically licenses DNA replication and mitotic machinery. Under exogenous auxin (2,4-D) stimulation, histone acetyltransferases (HATs) promote histone hyperacetylation (e.g., H3ac and H4ac), loosening the chromatin structure and reducing physical resistance ahead of the replication fork [14,23]. However, this structural looseness reduces the constraints on loop-extruding complexes, increasing the cell’s susceptibility to epigenetic instability, compartment mixing, and subsequent somaclonal variation [19,22]. Conversely, the second transition (embryogenic and shoot regeneration competence) requires a highly ordered, low-entropy chromatin configuration [28]. True embryogenesis requires more than simply “more organized” chromatin; it requires a developmentally competent state—able to support the coordinated activation and repression of large gene networks involved in establishing a new organism [30]. At the molecular level, this relies on a tightly tuned balance of epigenetic modifications. While direct genome-wide conformation capture (Hi-C) and chromatin accessibility assays (ATAC-seq) were beyond the scope of this cytological study, our structural findings align closely with emerging molecular models. Specifically, we propose a conceptual framework wherein Polycomb Repressive Complex 2 (PRC2) activity, H3K27me3 deposition, and auxin-mediated chromatin remodeling act as the molecular effectors governing the physical nuclear transitions observed in situ. Therefore, histone acetylation is required for initial chromatin relaxation, and Polycomb Repressive Complex 2 (PRC2) activity and histone methylation (H3K27me3) are essential to maintain the transcriptional silencing of embryogenic master regulators—such as LEAFY COTYLEDON2 (LEC2), BABY BOOM (BBM), and WIND1—in vegetative tissues [14,31]. Ectopic activation of totipotency requires overcoming this epigenetic repression and resetting the chromatin conformation [32]. When cells divide from a highly compact, organized state, the high-fidelity transmission of genetic and epigenetic information is ensured, preserving the developmental memory required for de novo organ patterning and avoiding the genomic instability typical of prolonged callus cultures [21,25].

### 3.4. Overcoming Recalcitrance: Practical Applications for Commercial Biofactories

Building upon the biophysical distinction between simple cell-cycle re-entry and true totipotent reprogramming, this structural framework provides a fresh perspective for understanding plant somatic recalcitrance [33]. Traditionally, tissue culture protocols attempt to overcome recalcitrance through empirical, trial-and-error adjustments of exogenous factors, including growth regulators, often with unpredictable outcomes [34,35]. Our findings suggest that recalcitrance may frequently reflect not a primary failure of biochemical hormone perception but rather a rigid topological constraint imposed by differentiated nuclear geometry and high structural resistance of chromatin [36]. From a translational perspective, while acknowledging that species-specific validation across broader crop systems remains necessary, establishing baseline 3D nuclear geometry (such as volume, sphericity, and compaction density) as a physical proxy offers a testable foundation for pre-culture explant diagnostics. By evaluating whether donor cell layers possess or can acquire permissive nuclear conformations prior to hormone exposure, cytological profiling could help identify competent explant tissues early, thereby reducing unnecessary culturing and streamlining micropropagation workflows. While high-resolution confocal laser scanning microscopy (CLSM) coupled with propidium iodide (PI) and RNase A treatments provides precise laboratory profiling, cost-effective alternatives can be adapted for industrial scales. Commercial biofactories can use standard optical microscopy with classical histological dyes that bind DNA and condensed chromatin structures, such as Giemsa, acetocarmine, DAPI, or Feulgen stains [37,38].

Quantitative image-processing software can then be used to calculate nuclear area, shape factor, and chromatin density parameters, establishing a “nuclear signature” of low entropy [23]. Explants or tissue layers failing to meet this geometric threshold can be discarded early, preventing unnecessary culturing [34].

Furthermore, understanding the thermodynamic barriers of the nucleus opens the door for targeted “epigenetic priming” in recalcitrant species [36]. Prior to standard hormone treatments, recalcitrant donor tissues could be treated with histone deacetylase inhibitors (such as Trichostatin A, TSA) or DNA methyltransferase inhibitors like 5-azacytidine (5-AzaC), as well as other small-molecule chromatin-remodeling agents [39,40]. While small-molecule epigenetic treatments were not directly tested in the present experimental setup, insights from other plant species support this biophysical strategy. Although 5-AzaC is primarily a DNA methyltransferase inhibitor, it also triggers significant secondary changes in genomic histone modification patterns and chromatin structure, making it a highly effective agent for altering global epigenetic landscapes [40,41]. Indeed, transitory treatments or early pulses with 5-AzaC, especially when combined with auxin, have been shown to drastically reduce global DNA methylation levels, alter nuclear chromatin architecture, and significantly improve somatic embryogenesis induction and embryo yield across diverse recalcitrant species [42,43,44]. This pre-treatment artificially relaxes the chromatin, lowering physical resistance and facilitating the precise, low-entropy epigenetic resetting required for de novo organogenesis [32,45]. Ultimately, incorporating nuclear architecture diagnostics and chromatin-priming strategies into biofactory workflows will drastically reduce somaclonal variation, accelerate micropropagation cycles, and unlock the totipotent potential of economically vital, recalcitrant crops.

### 3.5. Genome Stability and the Uncoupling of Proliferative History in Vitro

In planta, developmental organization strictly couples cell proliferation history with the requirement for genome stability. Cells contributing to long-term developmental lineages (such as stem cells and founder cells within the SAM and RAM) face strong evolutionary selection for genomic and epigenomic fidelity [23,25]. To ensure 100% protection against replication errors, these formative divisions occur slowly, allowing tightly regulated cell-cycle transitions within highly organized, low-entropy chromatin states [21]. By contrast, highly differentiated somatic cells can tolerate a different balance between proliferation speed and long-term genome maintenance. Because their divisions are rapid, with a shortened S-phase, they are more prone to replication-induced errors; however, because these somatic cells do not contribute to subsequent generations, these mutations are tolerated and ultimately lost [25,46]. Although the lack of a permanently segregated germline in plants makes this distinction less absolute than in animals, this protective dichotomy remains a cornerstone of plant development in vivo [47]. In vitro culture completely uncouples these normally coordinated properties, potentially leading to somaclonal variation [36]. During callus induction, rapidly proliferating somatic cells undergo extensive chromatin remodeling and lose their original tissue-enforced constraints [33,48]. When these rapidly dividing cells subsequently acquire regenerative competence, any genetic and epigenetic alterations accumulated during the disorganized, rapid proliferation phase become fixed and are propagated into the regenerated plant lineage [44,48]. This uncoupling represents a major biophysical source of somaclonal variation in micropropagation [46]. This genomic instability is particularly pronounced in suspension-cultured cells, which undergo rapid, relatively synchronized proliferation [48]. Throughout the culture cycle, fluctuations in auxin availability and cellular physiological age are accompanied by progressive, non-equilibrium changes in chromatin organization [22]. In this context, routine subculturing and medium renewal represent a drastic environmental reset. Nutrients and growth regulators are abruptly restored, forcing cells to enter a new culture cycle from chromatin states established during the preceding, depleted phase [48]. This mismatch between the inherited chromatin state and the new, aggressive proliferative stimulus destabilizes subsequent cell-cycle transitions and DNA replication licensing, triggering replication stress and driving subsequent genetic and epigenetic instability [22,46].

### 3.6. Epigenetic Regulation of Local Auxin Biosynthesis and the 3D Topological Barrier

Our model highlights that local auxin biosynthesis is tightly linked to developmental cell identity, forming a feedback loop with chromatin state [28]. Key auxin biosynthesis genes, such as TAA1/TAR and YUCCA, exhibit highly restricted spatial expression patterns in vivo and are themselves subject to strict epigenetic regulation [28]. For instance, developmental transitions involving YUCCA-dependent auxin production are accompanied by the dynamic deposition or removal of repressive chromatin marks, such as H3K27me3, mediated by the Polycomb Repressive Complex 2 (PRC2) [28,31]. Ectopic activation of totipotency regulators (such as LEC2) initiates somatic embryogenesis by remodeling this repressive chromatin and directly activating TAA1 and YUCCA expression, thereby resetting the somatic cell’s developmental fate [32,45]. We propose that these cell-type-specific modes of auxin biosynthesis are intimately associated with distinct, whole-nucleus 3D chromatin topologies that subsequently determine cell-cycle or regenerative behavior. This establishes a tripartite regulatory axis: the TAA/YUC transcriptional state to whole-nucleus 3D chromatin topology to the cell-cycle transition barrier [18,25]. In competent cells (such as the pericycle), local auxin biosynthesis maintains a compact, low-entropy chromatin architecture that acts as a permissive license for high-fidelity divisions [48]. Conversely, in differentiated somatic cells (such as *Arabidopsis* cortical cells), the silencing of these biosynthetic pathways is coupled to a highly compact heterochromatic state, creating a rigid topological barrier that blocks both division and reprogramming [21,25]. Overcoming this physical and epigenetic barrier requires a coordinated spatial reorganization of the genome, illustrating that somatic totipotency cannot be induced by exogenous auxin application alone without a corresponding structural reset of the nuclear architecture [19,22].

## 4. Materials and Methods

### 4.1. Plant Material and Growth Conditions

*Arabidopsis thaliana* (Col-0) and *Medicago sativa* (line A2) were used as plant material. Plants were grown as previously described [49,50]. Specifically, *Arabidopsis thaliana* plants were grown on TK1 [51] medium supplemented with 1% sucrose, and *Medicago sativa* on TK4 [49] medium supplemented with 2% sucrose in a growth room under a 16(8) light/dark cycle and a light intensity of 100 μmol/m^2^/s.

### 4.2. Root Treatments

Roots of 7-day-old *Arabidopsis* seedlings and 14-day-old *Medicago* plants were cut into segments of 3–4 mm in length and loaded onto the TK4 medium supplemented with 20 g/L sucrose, 1 mg/L 2,4-D, and 0.2 mg/L BAP. For the EdU label, root segments were incubated in the liquid medium, and EdU (10 μM final concentration) was added and incubated for 6 h. DIC images of explants after 4 days of cultivation were taken using a ZEISS Axiovert microscope (Oberkochen, Germany).

The Zeiss Stemi SV11 APO stereomicroscope (Oberkochen, Germany) was used for the overall general plan. After 7 days on these media, root segments were transferred to the TK4 medium supplemented with 1 mg/L BAP/0.2 mg/L NAA. They were incubated under light (100 μmol/m^2^/s).

### 4.3. Chromatin Imaging

Roots and root segments were fixed as described in [52]. Briefly, whole seedlings (4 to 6 days old) were fixed in 4% formaldehyde in MTSB for 30 min and washed three times with distilled H_2_O. Seedlings were incubated for 20 min in 100% methanol, followed by a gradual addition of H_2_O until the methanol concentration decreased to 5%. Roots were fixed in 4% FA in MTSB buffer and labeled with DAPI or double-labeled with PI and Calcofluor white. EdU incorporation detection was performed according to standard protocol as described [52].

Images were obtained with a confocal laser scanning microscope (LSM 5 DUO live Zeiss; Carl Zeiss, Oberkochen, Germany) with an LD LCI-Plan-Apochromat 925/0.8 DIC Imm Corr objective with glycerol as the immersion medium. For DAPI excitation, a diode laser (405 nm excitation) was used. PI was excited at 543 nm, and the emission was detected with a long-pass filter at 560. For EdU imaging, excitation was 488, and emission was 520–550 nm. Serial optical sections were reconstituted into 3D image stacks at a depth of 100 μm with in-plane (x–y) voxel extents of 0.15 μm and a section spacing of 0.9 μm in the z-direction. Emission was detected between 410 and 525 nm for DAPI and between 590 and 620 nm for PI. After immunolocalization, the seedlings were observed with a Zeiss LSM 510 laser scanning confocal microscope (Carl Zeiss AG, Oberkochen, Germany) equipped with UV, Ar, and He-Ne lasers.

### 4.4. Image Analysis

Cell types were detected based on the root coordinate system [53]. Chromatin analysis was performed as previously described [52]. Briefly, following 3D scanning, images were converted to TIFF files, and nuclei were segmented using the 3D Object Analysis plugin [54]. The resulting data were exported to Excel files for subsequent analysis. At least 12 nuclei of each type were analyzed in each biological replicate.

### 4.5. Root Protoplast Isolation and Cultivation

*Arabidopsis* root protoplasts were isolated and cultured as described [50]. *Medicago* root protoplasts were isolated as follows: roots were cut into 3–4 mm segments, incubated in enzyme solution (1% Cellulase, 1% Macerozyme in W5 solution, pH 5.2) for 10–12 h, filtered through 60 μm mesh, washed twice with W5, and cultured in T30 medium containing 1 mg/L 2,4,-D and 0.2 mg/L zeatin [49]. All procedures were performed essentially as described [49].

## 5. Conclusions and Predictive Outlook

In this study, we applied a systems-level biophysical framework linking cellular totipotency with nuclear organization, establishing 3D nuclear geometry as a quantifiable physical proxy for developmental plasticity. Our working hypothesis posits that simple cell-cycle re-entry and true totipotent reprogramming represent two distinct structural and epigenetic barriers. Crucially, the relaxed chromatin conformation sufficient to permit vegetative cell division is fundamentally uncoupled from, and insufficient for, driving true embryogenic competence, providing a testable biophysical foundation to predict and overcome somatic recalcitrance in plant biotechnology. We show that the ability of cells to re-enter and progress through the cell cycle correlates with the chromatin structural distance between cell types. In both species, pericycle nuclei display a relatively small structural distance from the reference proliferation-competent nuclear state, whereas *Arabidopsis* cortex nuclei show a substantially greater structural distance. Consistent with this relationship, *Medicago* cortex cells retain the capacity to re-enter the cell cycle but do not readily regenerate whole plants, whereas in *Arabidopsis* these properties are largely restricted to pericycle cells. Single-cell isolation and regeneration experiments could further test this relationship at the level of individual cells. Together, these observations suggest that chromatin structural distance between differentiated cell types and a proliferation-competent reference state may provide a quantitative indicator of their subsequent cell cycle and developmental competence. Our findings reveal that simple cell-cycle re-entry and true totipotent reprogramming represent two distinct physical and epigenetic barriers. Crucially, the relaxed chromatin conformation sufficient to permit vegetative cell division is fundamentally insufficient to guarantee embryogenic competence, providing a clear biophysical foundation to predict and overcome somatic recalcitrance in plant biotechnology.

## Figures and Tables

**Figure 1:**
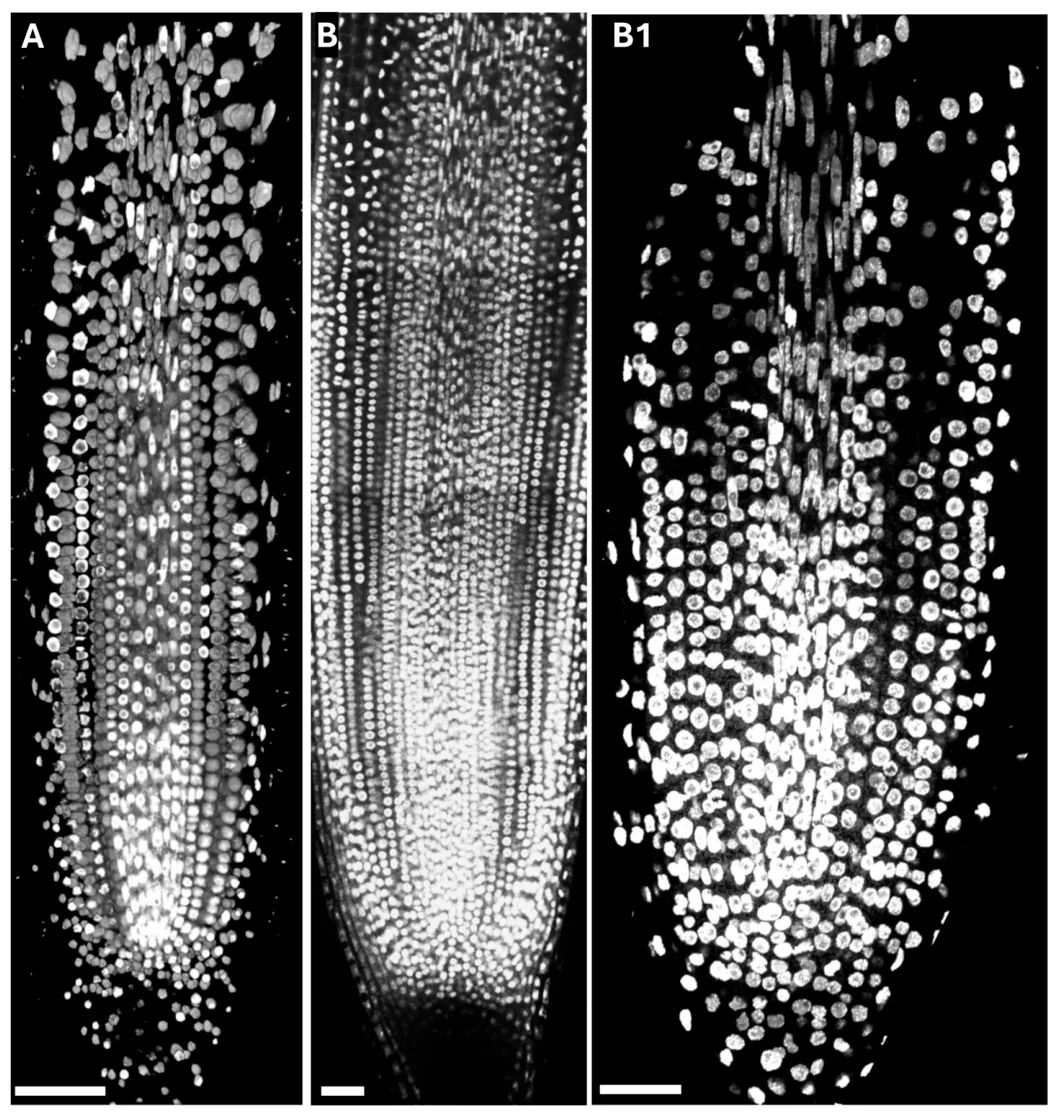
Nuclear architecture and chromatin organization in root tips up to the transition zone: (**A**) *Arabidopsis thaliana*; (**B**,**B1**) *Medicago sativa*. Scale bar = 50 µm. Roots were fixed and stained with propidium iodide following RNase A treatment to selectively visualize nuclear DNA geometry. Pericycle nuclei exhibit a stable, highly homogeneous geometric conformation, whereas cortical cell nuclei display pronounced structural heterogeneity and morphological variation.

**Figure 2:**
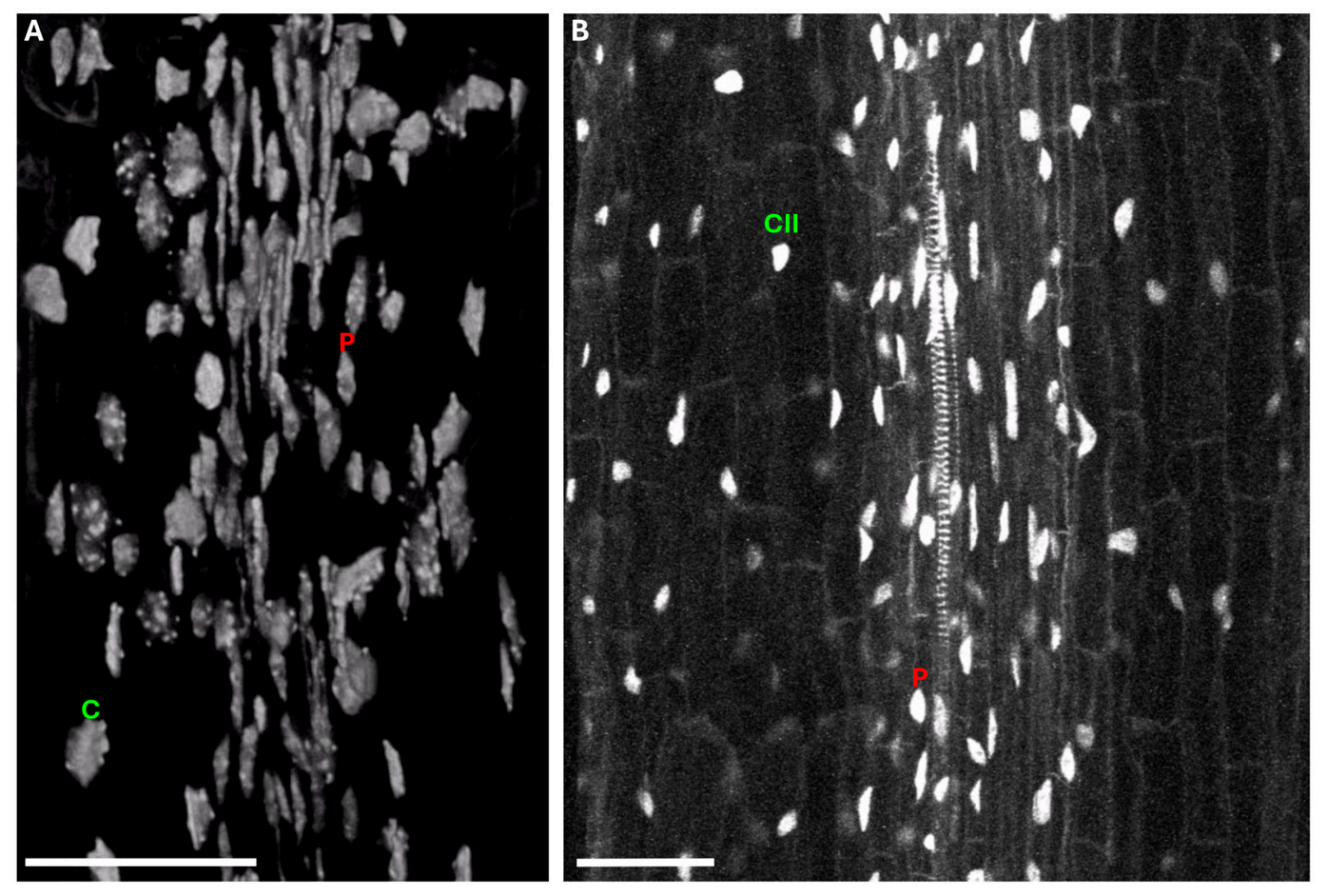
Three-dimensional (3D) radial gradients of nuclear architecture and cell division competence in mature roots. Confocal micrographs showing 3D reconstructions of the mature root zone in (**A**) *Arabidopsis thaliana* and (**B**) *Medicago sativa*. A pronounced radial gradient of nuclear morphology is established from the central vasculature to the outer epidermis, with *A. thaliana* exhibiting a significantly steeper gradient than *Medicago sativa*. This structural divergence correlates with cell-type-specific regenerative potential: mitotic re-entry is strictly restricted to pericycle cells in *A. thaliana*, whereas *M. sativa* shows active cell-cycle re-entry within the inner cortical layers. Scale bar: 50 μm. P—pericycle; C—cortex; CII—cortex 2.

**Figure 3:**
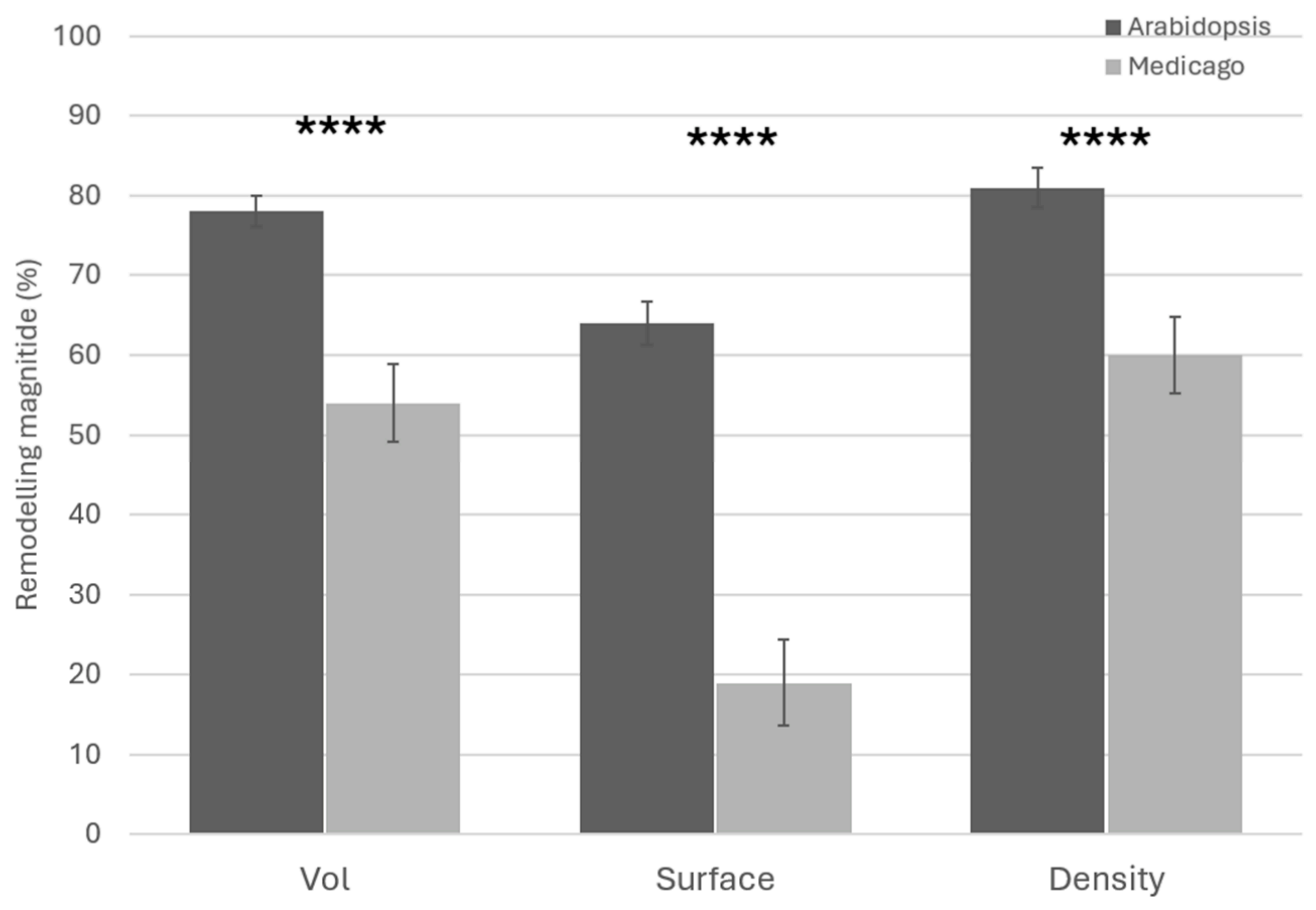
C-to-P nuclear remodeling magnitude in *Arabidopsis* and *Medicago*. Bars represent the percentage change from the mean cortex (C) value to the mean pericycle (P) value for nuclear volume, surface area, and integrated intensity; error bars indicate propagated SEM. Differences in C-to-P remodeling magnitude between species were assessed by bootstrap resampling of the individual C and P measurements (**** *p* < 0.0001).

**Figure 4:**
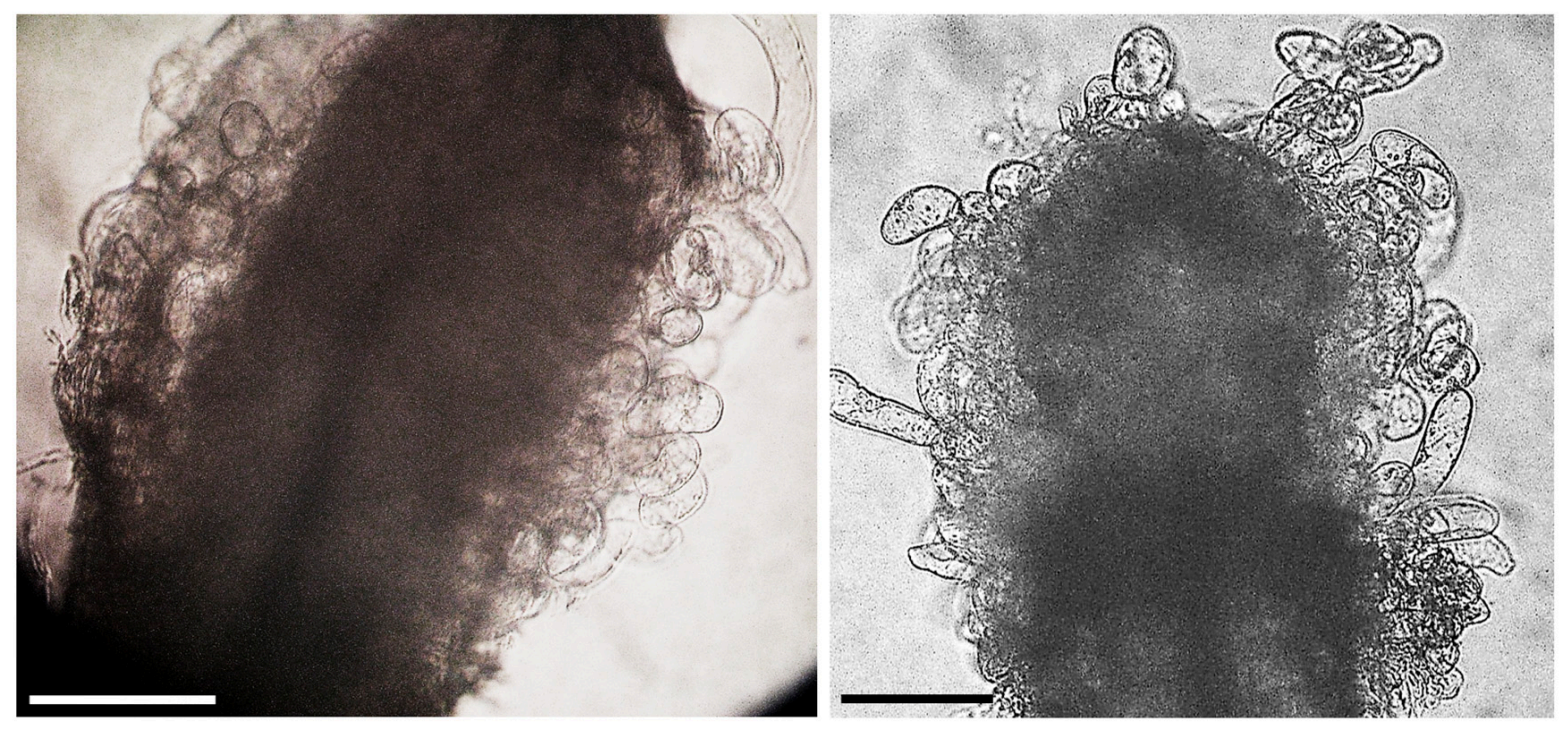
Auxin-induced outer cell type proliferation and soft callus formation in *Medicago sativa*. Representative micrograph of a *M. sativa* root explant showing rapid cell divisions and explosive callus initiation from the outer layers at 3–4 days after treatment with 1 mg/L 2,4-D. Scale bar = 100 µm.

**Figure 5:**
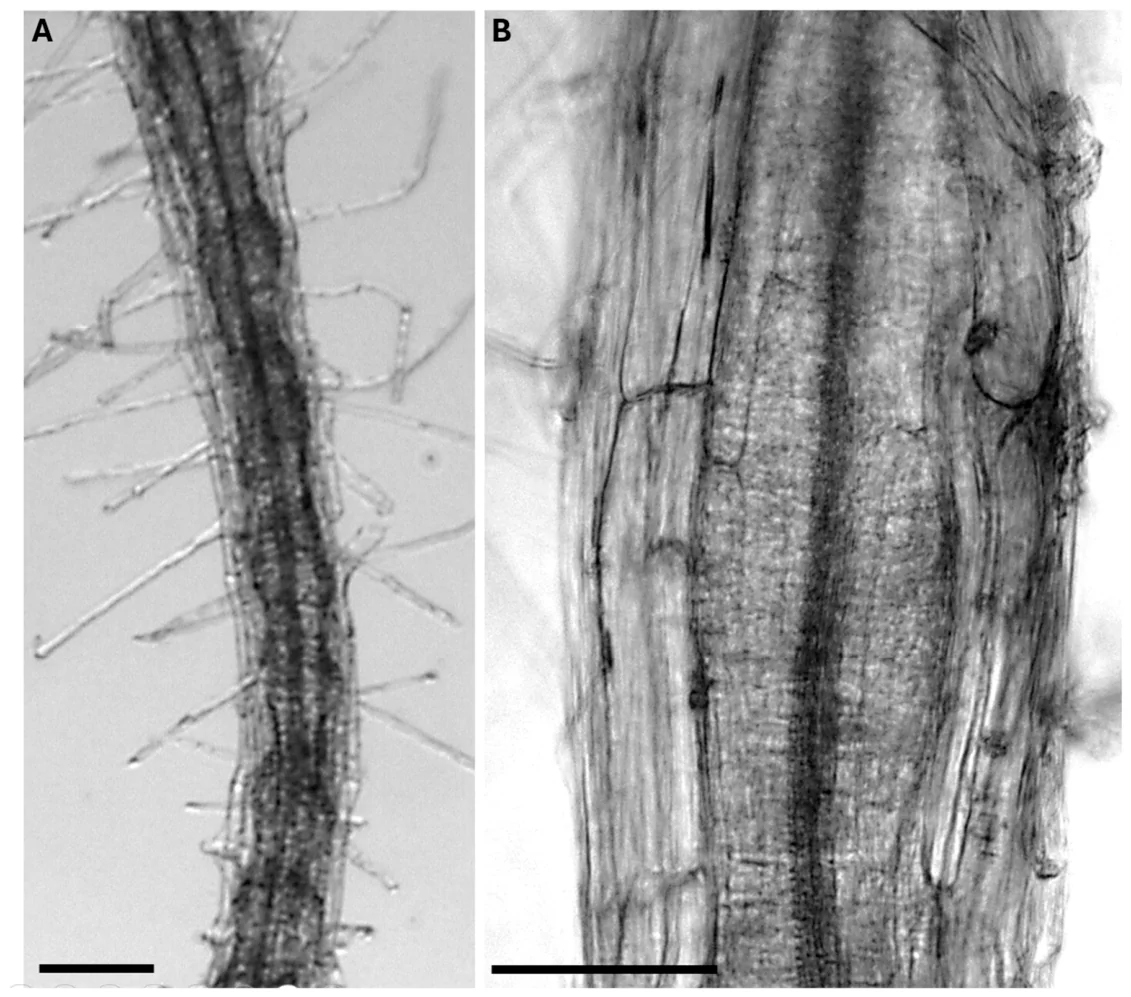
Tissue-specific cellular reactivation in *Arabidopsis thaliana* roots under exogenous auxin treatment. Confocal laser scanning micrographs of root segments cultured in the presence of 1 mg/L 2,4-D for 36 h, illustrating (**A**) a tissue-level overview of restricted mitotic reactivation and (**B**) high-magnification details of localized divisions. Scale bars: (**A**) 100 μm, (**B**) 50 μm. Note that cell division is strictly confined to the inner pericycle layer, while the outer cortical layers remain morphologically quiescent and insulated from auxin-induced mitotic re-entry.

**Figure 6:**
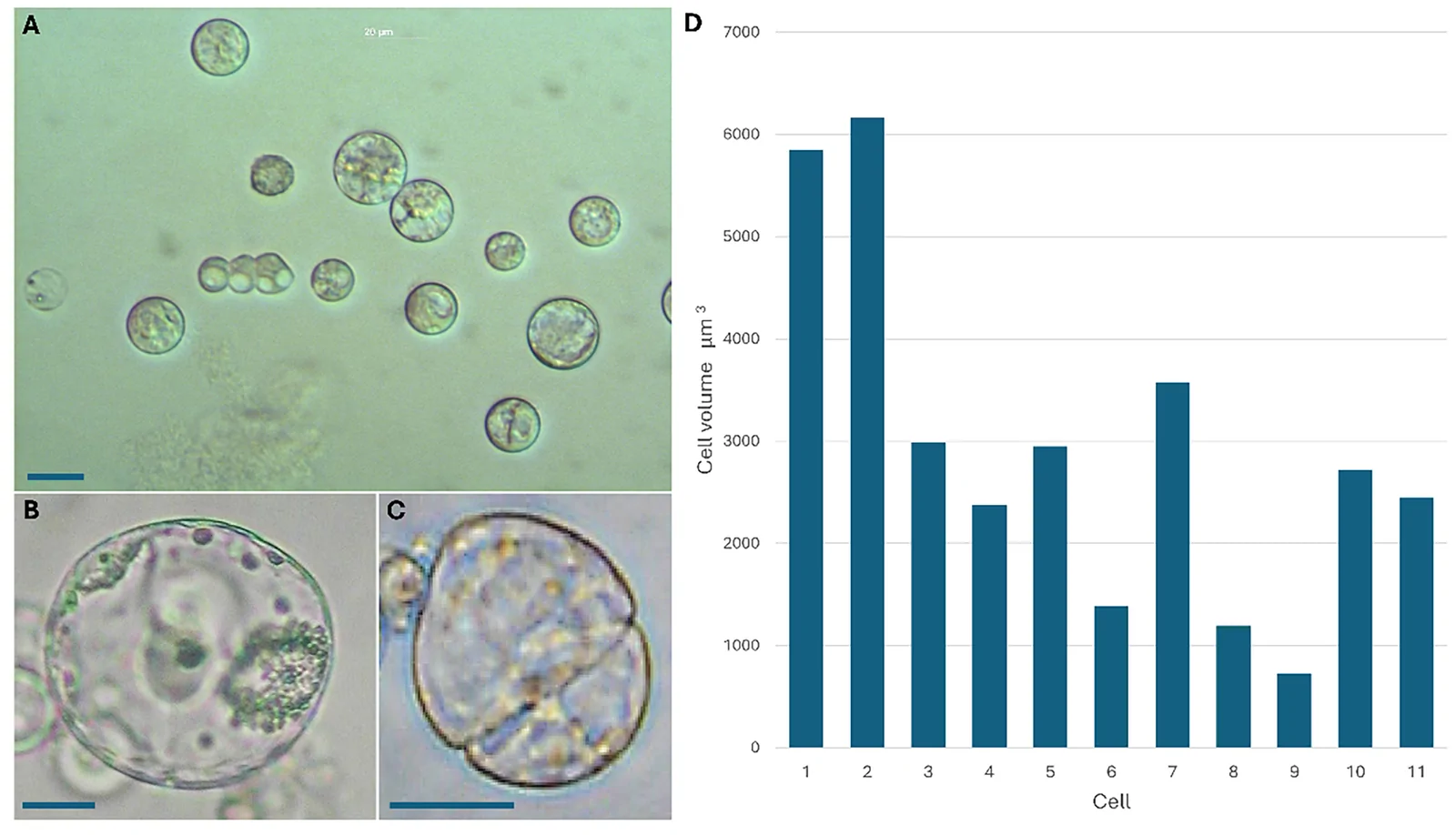
Isolation, purification, and morphometric characterization of tissue-specific protoplasts from *Arabidopsis thaliana* roots: (**A**) Enzymatic digestion of root tissues and subsequent density-gradient purification of viable protoplast populations. (**B**) Representative micrograph of isolated cortical protoplasts. (**C**) Micrograph of isolated pericycle-derived protoplasts. (**D**) Quantitative analysis of cell volume distribution in freshly isolated protoplast populations, highlighting the distinct physical and morphometric profiles.

**Figure 7:**
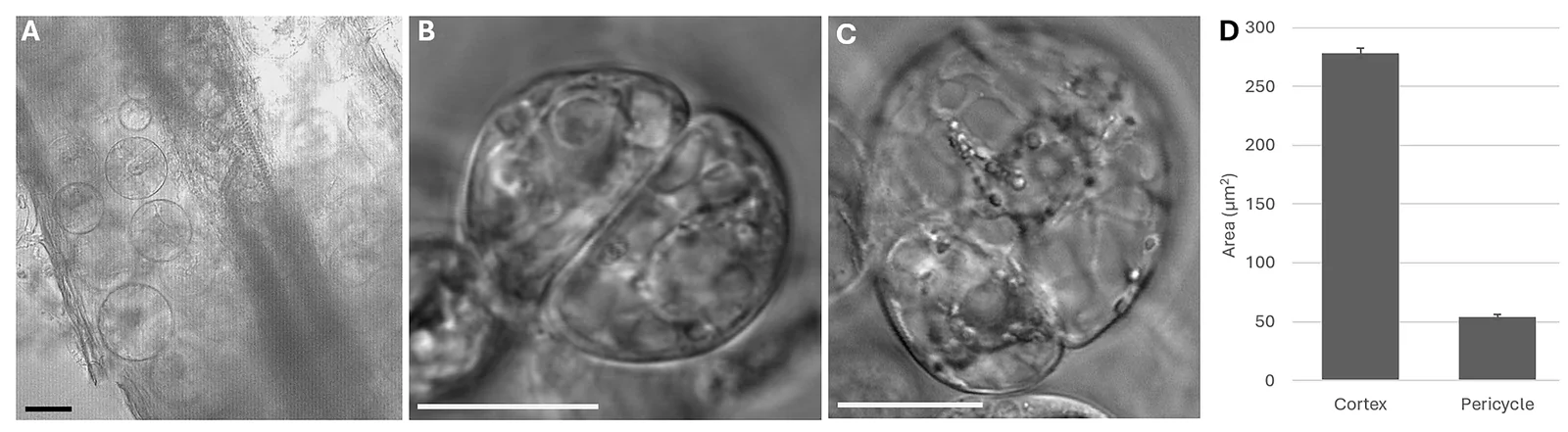
*Medicago sativa* root protoplasts: (**A**) root before isolation; (**B**) dividing pericycle; (**C**) dividing cortex; (**D**) cell area. Scale bars: 20, 5, and 10 μm.

## Data Availability

Data is contained within the article and Supplementary Materials.

## Supplementary Materials

The following supporting information can be downloaded at https://www.mdpi.com/article/10.3390/plants15213419/s1, Figure S1: Typical nucleus of *Medicago sativa* root tissue. A—reference nuclei (QC root); B—pericycle; C—cortex II. Scale bar—10 μm. Figure S2: Typical *Arabidopsis thaliana* root nuclei. A—reference (QC); B—pericycle; C—cortex. Scale bar A, B, C—10 μm. Figure S3: Spatial distribution of DNA replication in the *Medicago* root. Medicago seedlings were incubated in liquid medium containing 10 µM EdU for 6 h, fixed, and subjected to EdU detection. Cell walls were counterstained with calcofluor white. EdU-positive nuclei are shown in white, and cell walls are shown in green. Yellow arrows indicate representative EdU-positive nuclei in different root cell types. CI, cortex layer I; CIII, cortex layer III; En, endodermis. The image represents a projection of 15 central optical sections. Scale bar = 100 µm. Figure S4: Spatial distribution of DNA replication in the *Arabidopsis* root. *Arabidopsis* seedlings were incubated in liquid medium containing 10 µM EdU for 6 h, fixed, and subjected to EdU detection. EdU incorporation is shown in green and nuclei in magenta. Representative nuclei of different cell types are indicated: C, cortex; Ep, epidermis. The image represents a projection of 10 central optical sections. Scale bar = 50 µm. Figure S5: De novo plant regeneration from root segments of *Arabidopsis* root. Root segments were cultured on theTK4 medium supplemented with 1 mg/L 2,4-D/0.2 mg/L BAP for 7 days and thereafter transferred to the medium containing 1 mg/L BAP/0.1 mg/L NAA. Images were taken after 10 days. Scale bar = 1 mm.

## Abbreviations

The following abbreviations are used in this manuscript:

2,4-D	2,4-Dichlorophenoxyacetic acid
5-AzaC	5-Azacytidine
5mdC	5-Methyl-2′-deoxycytidine
AID	Auxin-Inducible Degron
BAP	6-Benzylaminopurine
BBM	BABY BOOM (Transcription Factor)
CDK	Cyclin-Dependent Kinase
CLSM	Confocal Laser Scanning Microscopy
CTCF	CCCTC-Binding Factor
CYCB	Cyclin B
DAPI	4′,6-Diamidino-2-phenylindole
DNA	Deoxyribonucleic Acid
FDA	Fluorescein Diacetate
GDM	Global DNA Methylation
HAT	Histone Acetyltransferase
HDAC	Histone Deacetylase
Hi-C	High-Throughput Chromosome Conformation Capture
H3K27me3	Histone H3 Lysine 27 Trimethylation
IAA	Indole-3-Acetic Acid
LEC1/2	LEAFY COTYLEDON 1/LEAFY COTYLEDON 2
LRP	Lateral Root Primordia
NAA	1-Naphthaleneacetic Acid
PI	Propidium Iodide
PRC2	Polycomb Repressive Complex 2
RAM	Root Apical Meristem
ROS	Reactive Oxygen Species
RNA-seq	RNA Sequencing
SAM	Shoot Apical Meristem
SE	Somatic Embryogenesis
SMC	Structural Maintenance of Chromosomes
TAA1/TAR	Tryptophan Aminotransferase of Arabidopsis 1/TAA1-Related
TSA	Trichostatin A
WIND1	WOUND INDUCED DEDIFFERENTIATION 1
WOX2	WUSCHEL-related Homeobox 2
WUS	WUSCHEL
YUCCA/YUC	YUCCA Auxin Biosynthesis Gene Family
