# Supplementary material for: Chromatin Organization and Nuclear Architecture as Indicators of Plant Cell Developmental Competence
Source: Plants (Basel). 2026 Nov 3;15(21):3419. doi: 10.3390/plants15213419 (PMC13765618; doi:10.3390/plants15213419)
Supplement: Supplementary file 1 [file plants-15-03419-s001.zip › plants-4641502-supplementary.pdf]

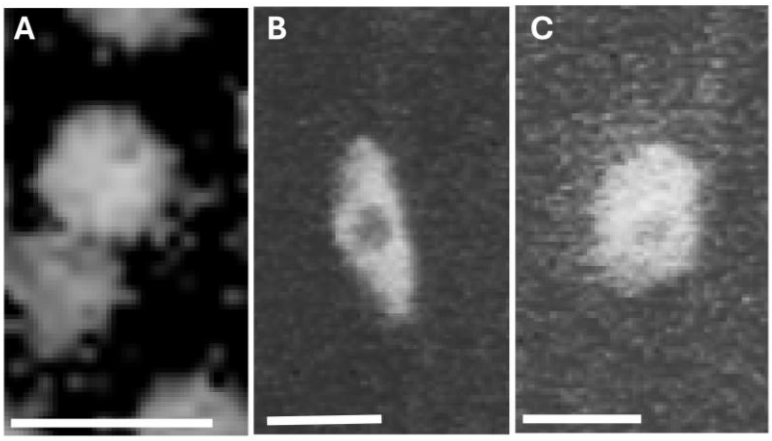

Figure S1. Typical nucleus of Medicago sativa root tissue. A-reference nuclei (QC root); B—pericycle; C—cortex II. Scale bar—10 µm.

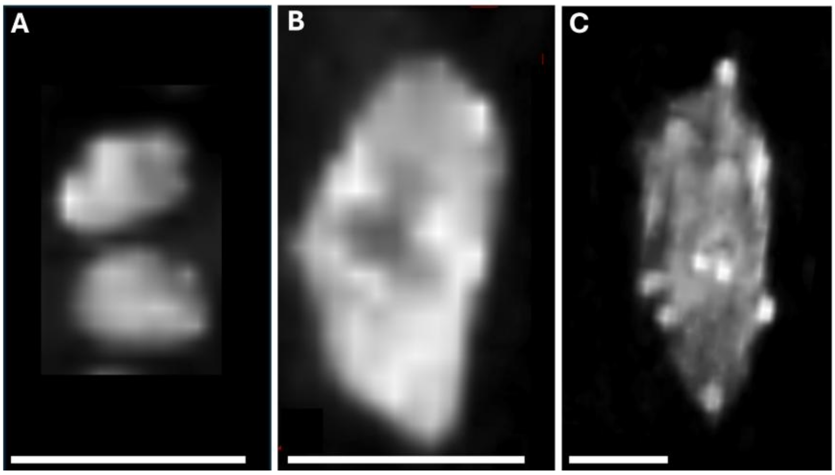

Figure S2. Typical Arabidopsis root nuclei. A -reference (QC); B—pericycle; C—cortex. Scale bar A, B,C—10 µm.

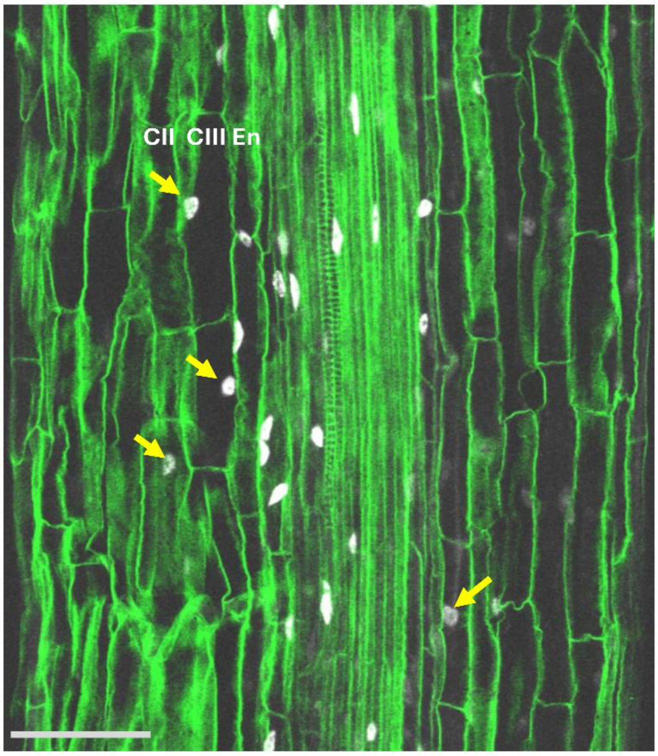

Figure S3. Spatial distribution of DNA replication in the Medicago root. Medicago seedlings were incubated in liquid medium containing 10 µM EdU for 6 h, fixed, and subjected to EdU detection. Cell walls were counterstained with calcofluor white. EdU-positive nuclei are shown in white, and cell walls are shown in green. Yellow arrows indicate representative EdU-positive nuclei in different root cell types. CI, cortex layer I; CIII, cortex layer III; En, endodermis. The image represents a projection of 15 central optical sections. Scale bar = 100 µm.

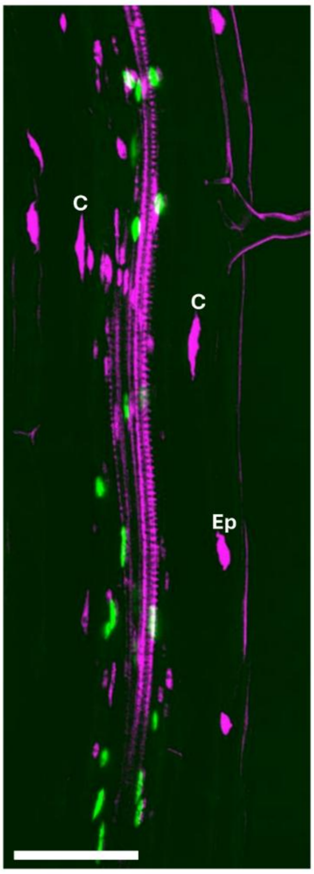

Figure S4. Spatial distribution of DNA replication in the Arabidopsis root. Arabidopsis seedlings were incubated in liquid medium containing 10 µM EdU for 6 h, fixed, and subjected to EdU detection. EdU incorporation is shown in green and nuclei in magenta. Representative nuclei of different cell types are indicated: C, cortex; Ep, epidermis. The image represents a projection of 10 central optical sections. Scale bar = 50 µm.

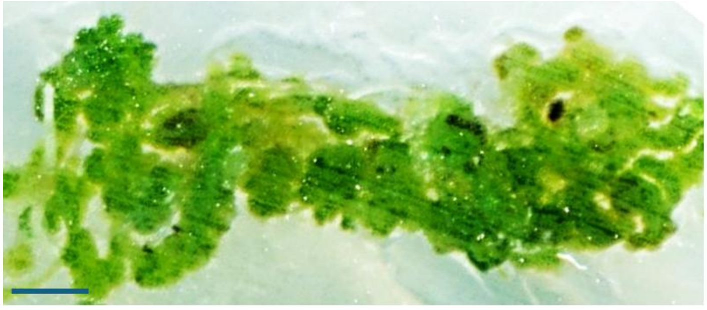

Figure S5. De novo plant regeneration from root segments of Arabidopsis root. Root segments were cultured on theTK4 medium supplemented with 1 mg/l 2,4-D/0,2 mg/l BAP for 7 days and thereafter transferred to the medium containing 1 mg/L BAP/0,1 mg/l NAA. Images were taken after 10 days. Scale bar = 1 mm.
